# From first to second wave: follow-up of the prospective COVID-19 cohort (KoCo19) in Munich (Germany)

**DOI:** 10.1186/s12879-021-06589-4

**Published:** 2021-09-08

**Authors:** Katja Radon, Abhishek Bakuli, Peter Pütz, Ronan Le Gleut, Jessica Michelle Guggenbuehl Noller, Laura Olbrich, Elmar Saathoff, Mercè Garí, Yannik Schälte, Turid Frahnow, Roman Wölfel, Michael Pritsch, Camilla Rothe, Michel Pletschette, Raquel Rubio-Acero, Jessica Beyerl, Dafni Metaxa, Felix Forster, Verena Thiel, Noemi Castelletti, Friedrich Rieß, Maximilian N. Diefenbach, Günter Fröschl, Jan Bruger, Simon Winter, Jonathan Frese, Kerstin Puchinger, Isabel Brand, Inge Kroidl, Andreas Wieser, Michael Hoelscher, Jan Hasenauer, Christiane Fuchs, the KoCo19 study group, Nikolaus Ackermann, Emad Alamoudi, Jared Anderson, Maxilmilian Baumann, Marc Becker, Franziska Bednarzki, Olimbek Bemirayev, Patrick Bitzer, Rebecca Böhnlein, Friedrich Caroli, Josephine Coleman, Lorenzo Contento, Alina Czwienzek, Flora Deák, Jana Diekmannshemke, Gerhard Dobler, Jürgen Durner, Ute Eberle, Judith Eckstein, Tabea Eser, Philine Falk, Manuela Feyereisen, Volker Fingerle, Otto Geisenberger, Christof Geldmacher, Leonard Gilberg, Kristina Gillig, Philipp Girl, Elias Golschan, Elena Maria Guglielmini, Pablo Gutierrez, Anslem Haderer, Marlene Hannes, Lena Hartinger, Alejandra Hernandez, Leah Hillari, Christian Hinske, Tim Hofberger, Sacha Horn, Kristina Huber, Christian Janke, Ursula Kappl, Antonia Keßler, Zohaib Khan, Johanna Kresin, Arne Kroidl, Magdalena Lang, Clemens Lang, Silvan Lange, Michael Laxy, Reiner Leidl, Leopold Liedl, Xhovana Lucaj, Fabian Luppa, Alexandra Sophie Nafziger, Petra Mang, Alisa Markgraf, Rebecca Mayrhofer, Hannah Müller, Katharina Müller, Ivana Paunovic, Michael Plank, Claire Pleimelding, Stephan Prückner, Elba Raimúndez, Jakob Reich, Viktoria Ruci, Nicole Schäfer, Benedikt Schluse, Lara Schneider, Mirjam Schunk, Lars Schwettmann, Andreas Sing, Alba Soler, Peter Sothmann, Kathrin Strobl, Jeni Tang, Fabian Theis, Sophie Thiesbrummel, Vincent Vollmayr, Emilia von Lovenberg, Jonathan von Lovenberg, Julia Waibel, Claudia Wallrauch, Julia Wolff, Tobias Würfel, Houda Yaqine, Sabine Zange, Eleftheria Zeggini, Anna Zielke, Thorbjörn Zimmer

**Affiliations:** 1 Institute and Outpatient Clinic for Occupational, Social and Environmental Medicine, University Hospital, LMU Munich, 80336 Munich, Germany; 2 Center for International Health (CIH), University Hospital, LMU Munich, 80336 Munich, Germany; 3 Comprehensive Pneumology Center (CPC) Munich, German Center for Lung Research (DZL), 89337 Munich, Germany; 4 Division of Infectious Diseases and Tropical Medicine, University Hospital, LMU Munich, 80802 Munich, Germany; 5 Institute of Computational Biology, Helmholtz Zentrum München-German Research Center for Environmental Health, 85764 Neuherberg, Germany; 6 Faculty of Business Administration and Economics, Bielefeld University, 33615 Bielefeld, Germany; 7 Core Facility Statistical Consulting, Helmholtz Zentrum München-German Research Center for Environmental Health, 85764 Neuherberg, Germany; 8 German Center for Infection Research (DZIF), partner site, Munich, Germany; 9 Center for Mathematics, Technische Universität München, 85748 Garching, Germany; 10 Bundeswehr Institute of Microbiology, 80937 Munich, Germany; 11 Interdisciplinary Research Unit Mathematics and Life Sciences, University of Bonn, 53113 Bonn, Germany

**Keywords:** COVID-19, SARS-CoV-2, Population-based cohort study, Sero-prevalence, Sero-incidence, ORCHESTRA

## Abstract

**Background:**

In the 2nd year of the COVID-19 pandemic, knowledge about the dynamics of the infection in the general population is still limited. Such information is essential for health planners, as many of those infected show no or only mild symptoms and thus, escape the surveillance system. We therefore aimed to describe the course of the pandemic in the Munich general population living in private households from April 2020 to January 2021.

**Methods:**

The KoCo19 baseline study took place from April to June 2020 including 5313 participants (age 14 years and above). From November 2020 to January 2021, we could again measure SARS-CoV-2 antibody status in 4433 of the baseline participants (response 83%). Participants were offered a self-sampling kit to take a capillary blood sample (dry blood spot; DBS). Blood was analysed using the Elecsys**®** Anti-SARS-CoV-2 assay (Roche). Questionnaire information on socio-demographics and potential risk factors assessed at baseline was available for all participants. In addition, follow-up information on health-risk taking behaviour and number of personal contacts outside the household (N = 2768) as well as leisure time activities (N = 1263) were collected in summer 2020.

**Results:**

Weighted and adjusted (for specificity and sensitivity) SARS-CoV-2 sero-prevalence at follow-up was 3.6% (95% CI 2.9–4.3%) as compared to 1.8% (95% CI 1.3–3.4%) at baseline. 91% of those tested positive at baseline were also antibody-positive at follow-up. While sero-prevalence increased from early November 2020 to January 2021, no indication of geospatial clustering across the city of Munich was found, although cases clustered within households. Taking baseline result and time to follow-up into account, men and participants in the age group 20–34 years were at the highest risk of sero-positivity. In the sensitivity analyses, differences in health-risk taking behaviour, number of personal contacts and leisure time activities partly explained these differences.

**Conclusion:**

The number of citizens in Munich with SARS-CoV-2 antibodies was still below 5% during the 2nd wave of the pandemic. Antibodies remained present in the majority of SARS-CoV-2 sero-positive baseline participants. Besides age and sex, potentially confounded by differences in behaviour, no major risk factors could be identified. Non-pharmaceutical public health measures are thus still important.

**Supplementary Information:**

The online version contains supplementary material available at 10.1186/s12879-021-06589-4.

## Background

The SARS-CoV-2 virus affected almost all nations within a few weeks. Given the nature of the virus, a large proportion of infected individuals present only mild symptoms or no symptoms at all. Therefore, population-based sero-prevalence studies are necessary to estimate the true prevalence of the infection in the population. Starting in March 2020, such sero-prevalence studies have been conducted in many countries, mostly during or after the first wave of the pandemic [1]. Depending on the serological test used, the type of sample drawn, the timing of the study, and the region, general population sero-prevalence ranged from < 0.1% in Brazil to well over 20% in the USA [2]. For the German context, we reported a sero-prevalence of 1.8% in Munich, sampled towards the end of the first wave in Germany [3].

Following the introduction of public health measures (lock-down including school closures) in March 2020 in Germany, the first wave of the pandemic was perceived as relatively mild with around 6000 cases registered in Munich during this period (Munich population ~ 1.5 Mio). Between June and October, public health measures were reduced, although physical distancing of 1.5 m between two persons, avoidance of mass events, and obligatory use of face masks, e.g. in restaurants and shops, were still required. Subsequently, officially registered monthly case numbers in Munich rose from 389 in June to 7181 in October 2020. A partial national lock-down was implemented on November 2nd, 2020. After a further rise in officially registered case numbers and COVID-19 related deaths, national lock-down measures were increased from December 16th, 2020 on, including closure of schools, shops (other than grocery and drug stores), restaurants, and hotels.

Given that asymptomatic and mildly symptomatic cases escape surveillance systems, prospective population-based cohort studies offer the chance to better understand the course of disease in the general population. They are independent of testing strategies and help to identify the population at risk over time. In addition, they provide an indication of population groups less well protected by public health measures. We therefore followed up the participants of the Munich COVID-19 cohort (KoCo19) to explore the SARS-CoV-2 antibody prevalence in the Munich general population at two time points: at the time the acute outbreaks happened and seven months later. In addition, we aimed at the identification of risk factors (demographic, social-economic, health status or individual risk behaviours factors) for acquiring SARS-CoV-2 infection defined by serology. The baseline study took place from April to June 2020, the questionnaire follow-up in summer 2020 and the 1st antibody follow-up was realised from early November 2020 to January 2021. On December 1st 2020 the KoCo19 cohort joined the ORCHESTRA (Connecting European Cohorts to Increase Common and Effective Response to SARS-CoV-2 Pandemic) project.

## Methods

### Study population and field work

#### Baseline SARS-CoV-2 antibody and questionnaire study

We described the baseline study in detail in [4]. In short, a random sample of the Munich population living in private households was drawn by random walk method. All household members older than 13 years were invited to provide a serum sample and to answer an online questionnaire. Serum samples were analysed for SARS-CoV-2 antibodies using the Elecsys**®** Anti-SARS-CoV-2 (Roche) test [5]. Field work for the baseline study took place between April 5th and June 12th, 2020.

#### Questionnaire follow-up

An online questionnaire covering risk behaviour, health related items, and psychosocial aspects (hereafter “behaviour questionnaire”) was offered from June 4th to October 31st, 2020 to all 5240 participants who did not withdraw from the study. In parallel, an online-questionnaire on leisure time behaviour was available (hereafter “leisure time questionnaire”). We split the questionnaire into two, because long questionnaires are less likely to be completed [6]. Participants recruited in April (May to June) 2020 received an invitation via e-mail on June 4th (June 25th) with subsequent reminders and telephone follow-ups. In total, 3400 participants completed the behaviour questionnaire and 1390 participants the leisure time questionnaire.

#### 1st SARS-CoV-2 antibody follow-up

On November 2nd 2020, we started the 1st antibody follow-up by sending out boxes with a self-sampling kit to take a capillary blood samples (dry blood spot; DBS) to the 5292 participants (2978 households) of the baseline study. Between baseline and follow-up, 77 participants withdrew from the study and were thus not contacted for the follow-up. Instructions for self-sampling were provided, including a video tutorial (https://www.youtube.com/watch?v=vpZUzuQV10E&feature=emb_title). Samples were collected using a barcode-labelled neonatal screening filter card (Euroimmun ZV 9701-0101) with circles indicating where the blood should be collected. Afterwards, participants should dry the filter card at least 12 h at room temperature, pack them in the sealable plastic pouch, place the plastic pouch into the prepaid envelope, and ship the envelope by mail to the laboratory. In case of handling difficulties, our telephone and e-mail hotline were available for any questions.

**Fig. 1:**
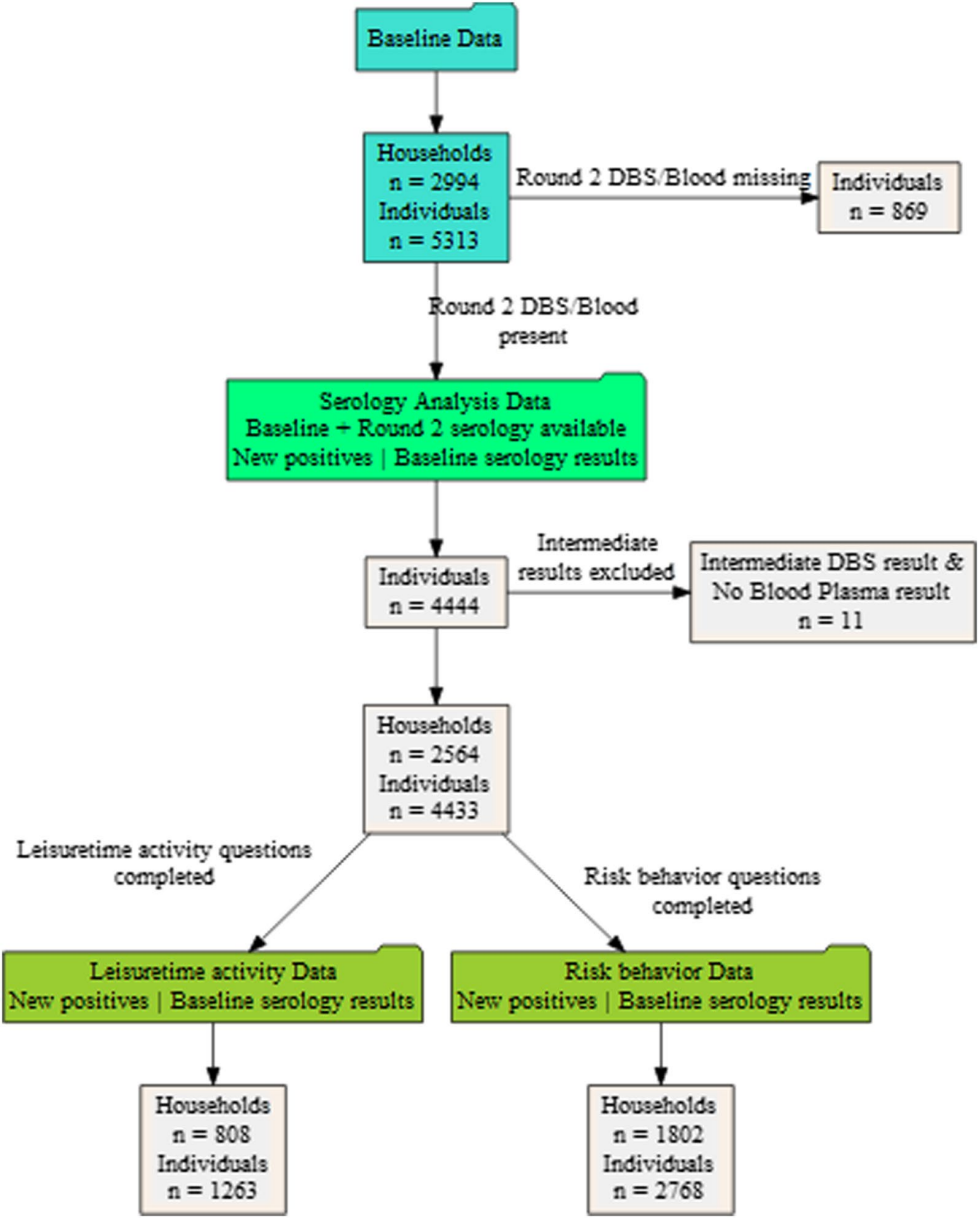
Flow chart of obtaining the study population. Light blue boxes indicate the total number of participants and households in the baseline study, the light green box refers to subjects included in the sero-prevalence analyses of the data, dark green boxes indicate analyses restricted to sero-incidence data for subjects with complete leisure time information (left) and risk behaviour data (right)

From November 2nd to January 31st, 2021, we received 4444 DBS samples from 2571 households (individual response 84%, household response 86%). Roughly half of the DBS samples (2372 of 4433; 54%) arrived at our laboratory within 1 week of mailing (November 2nd to November 8th). By week 2 (November 9th to November 15th), more than three quarters were received (3369 of 4433; 76%). Most of the remaining samples were turned in between week 3 (N = 372 from November 16th to November 22th) and week 4 (N = 343; November 23rd to November 29th). Few samples were received in December 2020 and January 2021 (N = 326; 7%). Participants not being able to collect a DBS on their own (N = 29) and those with intermediate results (N = 34, s. laboratory methods) were offered a full-blood test at our centre. For the latter group, this served to clarify the DBS result. However, 11 of the 34 participants with intermediate results in the DBS did not show up at our centre and thus had to be excluded from analyses, leaving 4433 subjects with baseline questionnaire, baseline serology and follow-up DBS data for the main analyses (Fig. 1).

### Questionnaire data

The following items were considered for the analyses presented in this paper:

Baseline individual questionnaire:

- Socio-demographics: age, sex (male, female), schooling (< 12 years, ≥ 12 years, in school), current job (employed, self-employed, not working (unemployed, retired, parental leave, sabbatical, students), others (voluntary social year, military service, part-time jobber, reduced working hours))
- Country of birth: Germany, others
- Smoking: current, ex, never smokers
- Chronic conditions: diabetes, cardiovascular diseases, autoimmune diseases, respiratory diseases (yes vs no)
- General health: “In general, how would you rate your health” assessed on a five point Likert scale from poor to excellent. As very few participants reported “poor”, the poor and fair category were combined.

Baseline household questionnaire:

- Household size: 1, 2, 3–4, > 5 inhabitants
- Household income: ≤ 2500 €, 2500– ≤ 4000 €, 4000– ≤ 6000 €, > 6000 €
- Living area per inhabitant: ≤ 30 sqm, 30– ≤ 40 sqm, 40– ≤ 55 sqm, > 55 sqm
- Household type: single, couple, family, others (shared apartments by e.g., students, subleasing, and assisted accommodation)
- Housing type: building with 1–2 apartments, 3–4 apartments, ≥ 5 apartments

Follow-up questionnaire:

- Self-estimated health-related risk taking behaviour (10-level Likert scale from “not at all risk tolerant” to “very risk tolerant”): Dichotomised into not high (≤ 5, Quartile 3) and high self-estimated health-related risk taking behaviour (> 5)
- Personal contacts: Five questions on places of personal contacts outside the own household during the two weeks before answering the questionnaire (meeting people, grocery shopping, shopping, use of public transport, work outside home), each assessed on a 5-level Likert scale: not at all (= 1); once per week (= 2); 2–4 times per week (= 3); 5 times per week (= 4); more often (= 5). Places of personal contacts were multiplied by frequency of contacts (0 contacts (= 0), 1 contact (= 1), 2–4 contacts (= 2) and 5 + contacts (= 3)) and summed up, resulting in a score ranging from 0 to 25. The score was dichotomised into lower number of personal contacts (≤ 8, Median) and higher number of personal contacts (> 8). The score was dichotomised into non-high leisure time activities (≤ 11, Quartile 3) and high leisure time activities (> 11).
- Number and intensity of leisure time activities before the pandemic (in February 2020): For that time, 16 activities assessed on a 5-level Likert scale from “never” (= 0) to “very often” (= 4): visit family member; visit friends; going out with friends; attend a party, festival, bar, pub or disco; go to the cinema; attend a theatre, opera or ballet performance; work out in a gym; visit a swimming pool; visit a sauna; skiing; train for a team sport or take part in sporting competitions; watch a sports game or event live outdoors; watch a sports game or event live indoors; worship attendance; play an instrument in an orchestra; sing in a choir. Activities were multiplied by the Likert scores and summed up resulting in a score from 0 to 64.
- Number and intensity of leisure time activities two weeks prior to the follow-up questionnaire: The score for leisure time activities at follow-up was built the same way as the score for leisure time activities before the pandemic. However, the number of leisure time activities was only seven at that time as many activities were not possible due to the restrictions related to the pandemic: visit family member; visit friends; going out with friends; visit a swimming pool; worship attendance; play an instrument in an orchestra; sing in a choir. Therefore, the resulting score only ranged from 0 to 28. The score was dichotomised into non-high leisure time activities at follow-up (≤ 5, Quartile 3) and high leisure time activities (> 5).

### Laboratory method and cross-validation with blood samples

Filter paper cards were further processed if at least two of the five circles on the card were completely soaked with blood. Valid samples were stored at 4 °C until analysis. Before analysis, filter paper cards were equilibrated to room temperature and three blood-soaked smaller circles (diameter 3.2 mm) of each filter paper card were automatically punched into a 96-wells plate (Panthera-Puncher™ 9, PerkinElmer). After elution, samples were transferred to a Cobas e801 module (Roche) compatible sample micro cup (Roche, 05085713001) for analysis using the Elecsys**®** Anti-SARS-CoV-2 assay (Roche). Based on our validation study, DBS samples were considered positive if SARS-CoV-2 antibody levels were ≥ 0.12. Samples with SARS-CoV-2 antibody levels in the range between 0.09 and 0.12 were considered intermediate, and subsequently confirmed by plasma samples (s. Study population and field work). All other samples were considered negative. Compared to full blood samples, sensitivity of the DBS method was 99.2% and specificity 98.7%. Details of the laboratory methods are described in [7].

### Statistical analyses

All statistical analyses were performed using the statistical software R (version 4.0.3, R Development Core Team, 2020).

The SARS-CoV-2 sero-prevalence was estimated primarily based on the DBS test results of the study participants applying the classification as described above (Laboratory methods). If the DBS test yielded an intermediate result, we considered the result of the full blood sampling. As described in [5], an optimised cut-off of 0.4218 for the full blood sampling was used to predict SARS-CoV-2 sero-positivity with an estimated specificity and sensitivity of 99.7% and 88.6%, respectively (with regard to PCR test results considered as ground truth). We used these estimates to adjust the prevalence for the imperfect test performance [8]. The specificity and sensitivity of DBS with regard to full blood samples being very high, additional adjustment was omitted (Additional file 1: Appendix Text and Table S1).

The prevalence (adjusted or unadjusted for the specificity and the sensitivity of the test) was calculated in two different ways: including the information from the sampling design of the cohort [3] via the use of a weighting scheme, or without it. To account for the sampling design, the sampling weights computed at baseline (inverse of the probability of each individual to be included in the sample) were used for the follow-up analysis. These sampling weights were corrected for the attrition observed at follow-up by modelling the underlying non-response mechanism and estimating probabilities of response for each unit. Ten response homogeneity groups (where we assumed the non-response to be completely at random, [9]) were created using the deciles of the estimated probabilities of response. These weights adjusted for the non-response were calibrated [10] on updated information from the Munich population (at 31.12.2020) in order to mirror the age, sex, country of birth, presence of children in the household and single member household structures. Moreover, to correct the sample for the loss of positive cases at follow-up, the sampling weights were calibrated on the estimated number of positive cases at baseline. Weighted prevalence estimates were calculated using these calibrated weights, and the associated 95% confidence intervals were computed based on variance estimators based on linearization [10] and residual [10, 11] techniques. These variance estimates were computed in order to account for every step in the selection process of the units, i.e., V = V1 + V2 with V1 the variance due to the sampling design and V2 the one due to the non-response [12]. For unweighted prevalence estimates, confidence intervals were determined by using a nonparametric cluster bootstrap procedure that accounts for household clustering [13]. To that end, 5000 bootstrap datasets were generated each by sampling n_h_ households with replacement from the original sample of n_h_ households. The sero-prevalence was estimated in each bootstrap sample and the 2.5 and 97.5 percentiles of the resulting 5000 estimates defined the 95% confidence intervals.

To analyse spatial clustering, we considered the mean within-cluster variance of the binary test results, with cluster variables being households, buildings, and geospatial clusters of different sizes. We performed a non-parametric approximate permutation test with 10,000 random permutations of cluster assignments. To account for household clustering, only full households were permuted when considering buildings and geospatial clusters [14]. In addition to this, we performed borough level sero-prevalence mapping using Conditional Auto Regressive Models which account for the spatial autocorrelation among neighbouring boroughs by using random effects. This allowed us to investigate if sero-prevalence was associated with the population density or not, as well as obtaining Borough/District level estimates within the city of Munich (Additional file 1: Appendix 2) [15–18].

We used generalised linear mixed models (GLMMs using the logit link function) to analyse the association between potential risk factors and SARS-CoV-2 sero-positivity at 1st follow-up, with a random effect for households to account for within household clustering of the data. Odds Ratio estimates and the corresponding confidence intervals were obtained applying a Bayesian framework with uniform priors on the regression estimates using the brms (Bayesian Regression Models using 'Stan') package in R [19, 20]. To account for missing data in covariates, we used the Joint Analysis and Imputation of Incomplete Data Framework (JointAI) in R for sensitivity analyses [21, 22]. In these sensitivity analyses, broad normal priors with mean zero and standard deviation 100 were used. The regression estimates were adjusted for the SARS-CoV-2 serology results at baseline, the time elapsed since baseline visit, age, and sex of the individual. Essentially, this adjustment for baseline positivity allowed us to obtain risk factors associated with newly incident cases within our cohort over and above the baseline positives.

To explore the importance of behavioural factors and leisure-time activities for the incidence of infection between baseline and follow-up, we used data of the 1st questionnaire follow-up combined with the DBS results. For these analyses, we included information of 2768 participants who responded to the behaviour questionnaire and had serology results; for the leisure time activities, we had questionnaire information for 1263 persons with serological results. Due to the large proportion of missing questionnaire data, we restricted these analyses to complete data and aggregated at the levels of the outcome variables. We analysed the incidence of new SARS-CoV-2 infections (as binomial outcome for proportions) between baseline and follow-up stratified for risk behaviour, leisure time activities, sex and age, using the count of new positives among the observed. Similar models were also applied to evaluate the association of the population densities at the constituency level and the trend in sero-prevalence estimates using aggregated data.

## Results

### Non-responder analysis

Follow-up participants compared to participants lost to follow-up were more likely to be between 35 and 79 years old, born in Germany (84% vs. 74%), and to have a higher socio-economic status (Table 1). The latter was indicated, e.g., by level of education, household income, living area, and type of building. In addition, the sero-prevalence of SARS-CoV-2 at baseline was lower among follow-up participants (1.6%) compared to baseline only participants (2.6%; Table 1). These losses of positive cases at follow-up led to an underestimation of the total number of people tested positive at baseline in Munich (22,064 vs. 25,900 using all participants at baseline). To correct for this attrition bias, the weights at follow-up were calibrated on the estimate of positive cases at baseline, in addition to the other margins used to mirror the Munich structure.

**Table. 1:** Descriptive data of the KoCo-19 follow-up participants in comparison to participants only taking part in the baseline study (“Losses to follow-up”)

Variable	Categories*	N	n_Missing_	Losses to follow-up (N = 880)		Follow-up participants (N = 4433)		p^#^
				n	%	n	%	
	Total	5313	0	880	16.6	4433	83.4	
Sex	Female	2766	0	446	50.7	2320	52.3	0.38
Age	0–19	267	0	55	6.3	211	4.8	< 0.001
(years)	20–34	1346		306	34.8	1040	23.5	
	35–49	1542		271	30.8	1271	28.7	
	50–64	1306		140	15.9	1166	26.3	
	65–79	676		77	8.8	599	13.5	
	80+	176		31	3.5	145	3.3	
Birth country	Germany	3999	465	478	73.9	3521	83.8	< 0.001
Level of education	Student	100	701	20	3.2	80	2.0	0.01
	< 12 years	1386		211	33.8	1175	29.5	
	≥ 12 years	3126		394	63.0	2732	68.5	
Occupationally active	Yes	3935	470	522	80.8	3413	81.3	0.75
Smoking status	Never smoker	2540	487	323	50.0	2217	53.0	0.007
	Ex-smoker	1411		177	27.4	1234	29.5	
	Current smoker	875		146	22.6	729	17.5	
General health	Excellent	798	466	112	17.3	686	16.4	0.20
	Very good	2126		274	42.3	1852	44.1	
	Good	1717		224	34.6	1493	35.5	
	Not good	206		37	5.7	169	4.0	
Respiratory allergies	Yes	1379	540	187	29.2	1192	28.8	0.85
Diabetes	Yes	208	504	41	6.4	167	4.0	0.009
CVD	Yes	892	513	91	14.2	801	19.3	0.002
Obesity	Yes	279	521	31	4.9	248	6.0	0.28
Household type	Single	680	494	92	14.4	588	14.1	< 0.001
	Couple	1705		174	27.2	1531	36.6	
	Family	1953		279	43.6	1674	40.1	
	Others	481		95	14.8	386	9.2	
Household income	≤ 2500	593	1636	92	20.3	501	15.5	0.02
(Euro)	2501–4000	817		111	24.4	706	21.9	
	4001–6000	1176		133	29.3	1043	32.4	
	6000 +	1091		118	26.0	973	30.2	
Living area/inhabitant	≤ 30	1702	513	270	42.5	1432	34.4	< 0.001
(sqm/individual)	31–40	1213		175	27.5	1038	24.9	
	41–55	988		99	15.6	889	21.3	
	55 +	897		92	14.5	805	19.3	
Building type	1–2	1433	0	170	19.3	1263	28.5	< 0.001
(No of apartments)	3–4	354		47	5.3	307	6.9	
	5 +	3519		663	75.3	2856	64.4	
	Others	7		0	0.0	7	0.2	
Baselinesero-prevalence	Positive	93		23	2.6	70	1.6	0.047

*For category definitions please see the methods section of the article

^#^p-values were obtained by Chi Square Test using 10,000 replicates for variables with more than 2 categories. For variables with only 2 categories, p-values were obtained by Fisher Exact Test

### SARS-CoV-2 sero-prevalence over time

**Fig. 2:**
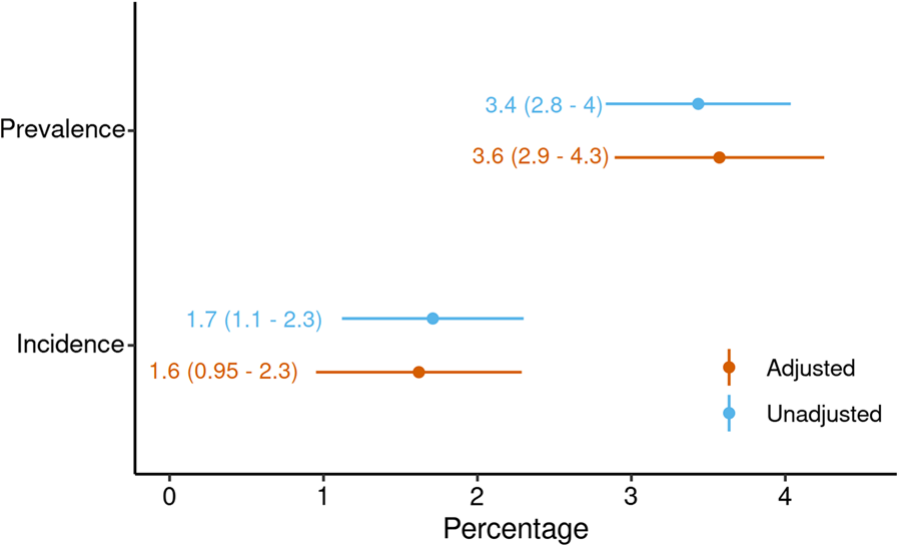
Weighted sero-prevalence and sero-incidence in % at follow-up adjusted (orange) and unadjusted (blue) for test specificity and sensitivity. The unadjusted weighted sero-prevalence was 3.4%, the relative number of new cases between baseline and follow-up 1.7%. Adjustment only slightly changed the unadjusted results

**Fig. 3:**
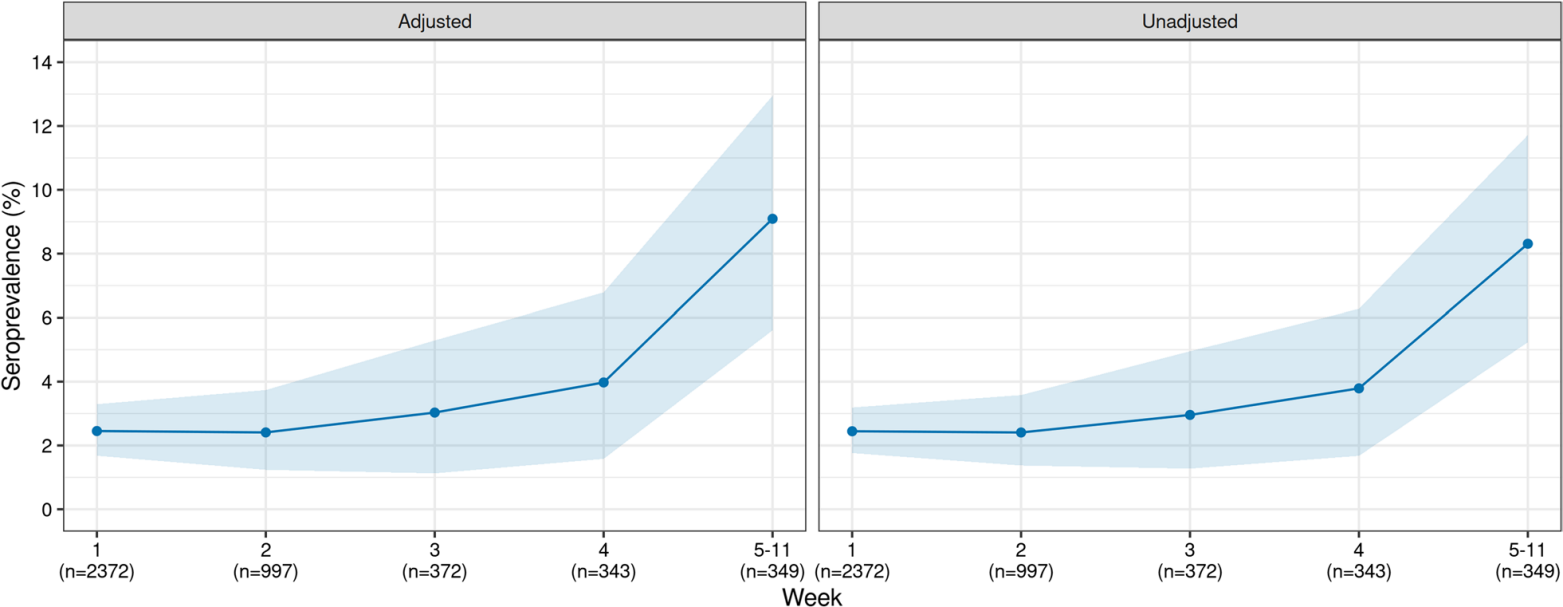
For sensitivity and specificity adjusted (left) and unadjusted (right) SARS-CoV-2 sero-prevalence over the follow-up period. The 95% confidence intervals for the weekly sero-prevalence were based on the 2.5 and 97.5 percentiles from 5000 repetitions of a cluster bootstrap that accounts for within household clustering. The estimates do not account for sample weights. The estimation without accounting for within-household clustering but considering sample weights produced similar trends (Additional file 1: Figure S1). A slight increase of sero-prevalence is indicated from the first to the fourth week of follow-up. The huge increase from week 4 to weeks 5–11 has to be taken with caution, as during these weeks, participants with intermediate results in the DBS (of whom 50% turned out to be positive in plasma sampling) were be retested by plasma-sampling during this time interval. The upward trend without these participants is shown in Additional file 1: Figures S1 and S2

The overall weighted and adjusted (for specificity and sensitivity) SARS-CoV-2 sero-prevalence at follow-up was 3.6% (95% CI 2.9–4.3%; Fig. 2). The overall unweighted and adjusted sero-prevalence was 3.1% (95% CI 2.5–3.8%), increasing from 2.5% (95% CI 1.7–3.3%) in the first week of November to 4.0% (95% CI 1.6–6.8%) in the last week of November (Fig. 3). About half of the participants with intermediate result in the DBS test had a positive test result when considering the plasma sample. As plasma samples were collected in December and January, the prevalence estimates in the latest weeks were artificially high. Yet, the overall upward trend remained after excluding the participants with intermediate DBS result (Additional file 1: Figures S1, S2).

Most participants who were SARS-CoV-2 sero-positive at baseline continued to be sero-positive at follow-up (64 out of 70 sero-positive subjects at baseline; Additional file 1: Table S3). The weighted and adjusted SARS-CoV-2 sero-incidence (negative at baseline, positive at follow-up) was estimated at 1.6% (95% CI 0.95–2.3%) (Fig. 2).

### Geospatial distribution of SARS-CoV-2 sero-prevalence

**Fig. 4:**
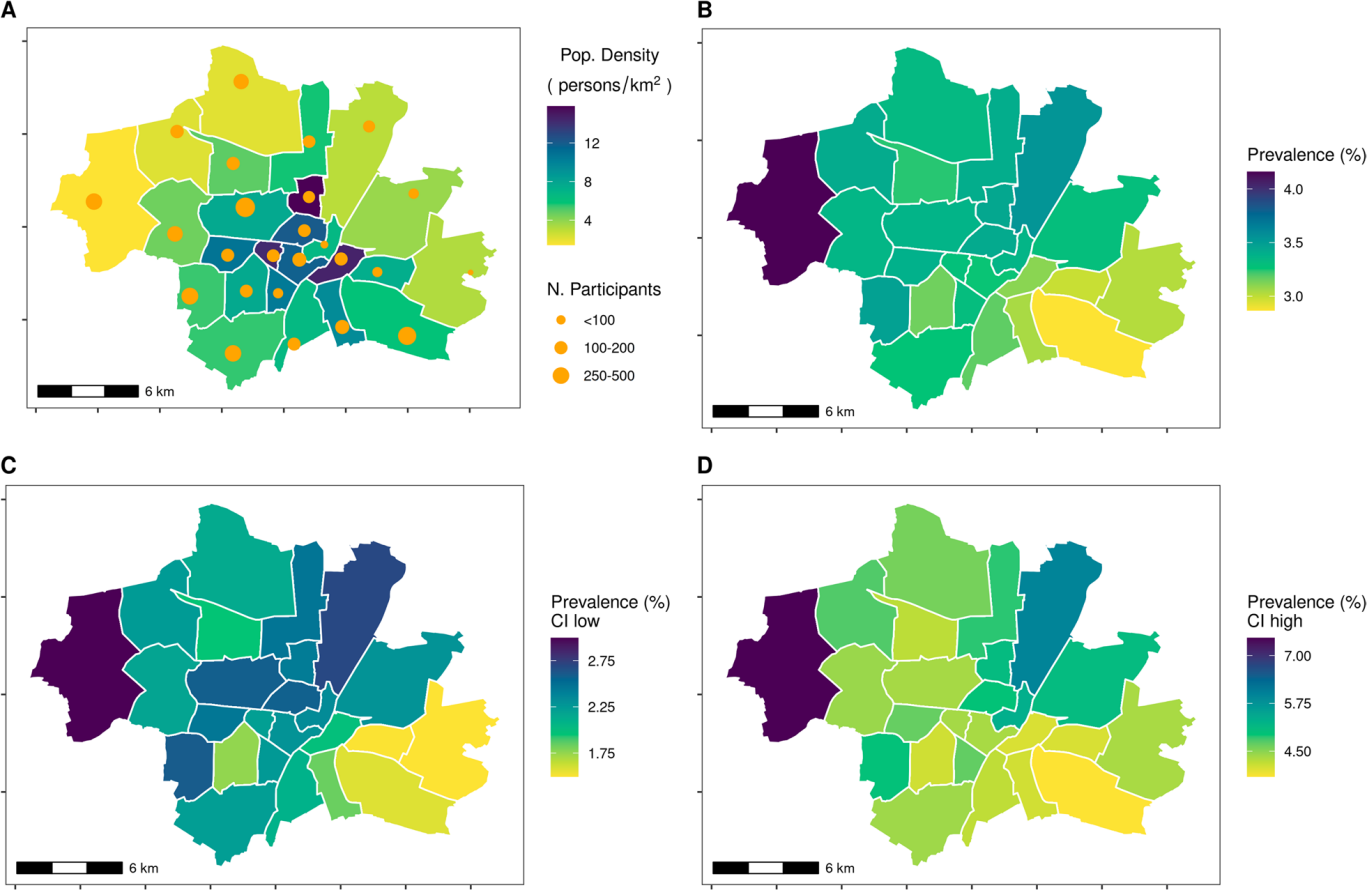
Geospatial distribution of the crude SARS-CoV-2 sero-positivity across boroughs in Munich. **A** Population density (taken from https://simple.wikipedia.org/wiki/Boroughs_of_Munich) and number of participants in each city borough; **B** Weighted sample based SARS-CoV-2 sero-prevalence; **C** Lower 95% confidence bounds of the weighted SARS-CoV-2 sero-prevalence; **D** Upper 95% confidence bounds of the weighted SARS-CoV-2 sero-prevalence. The sero-positivity varied slightly across the boroughs (as indicated by different colours), however, differences did not reach statistical significance

Looking at the geospatial distribution of SARS-CoV-2 sero-prevalence by Munich city boroughs (Fig. 4), an increase was visible from the South-East to the North-West of the city, although these differences were rather small. The estimates of Moran’s I for spatial autocorrelation was 0.015 using the continuous distance based spatial neighbourhood matrix resulting in a p-value of 0.304, and hence was not statistically significant. Using a binary spatial neighbourhood matrix, the estimated Moran’s I was 0.025 with a corresponding p-value of 0.269—thereby the conclusion remained unchanged. We also took population density as a potential risk factor into account and could not find any statistically significant association between population density in the constituency and SARS-CoV-2 sero-prevalence (Additional file 1: Figure S3).

### Risk factors for SARS-CoV-2 sero-prevalence

**Fig. 5:**
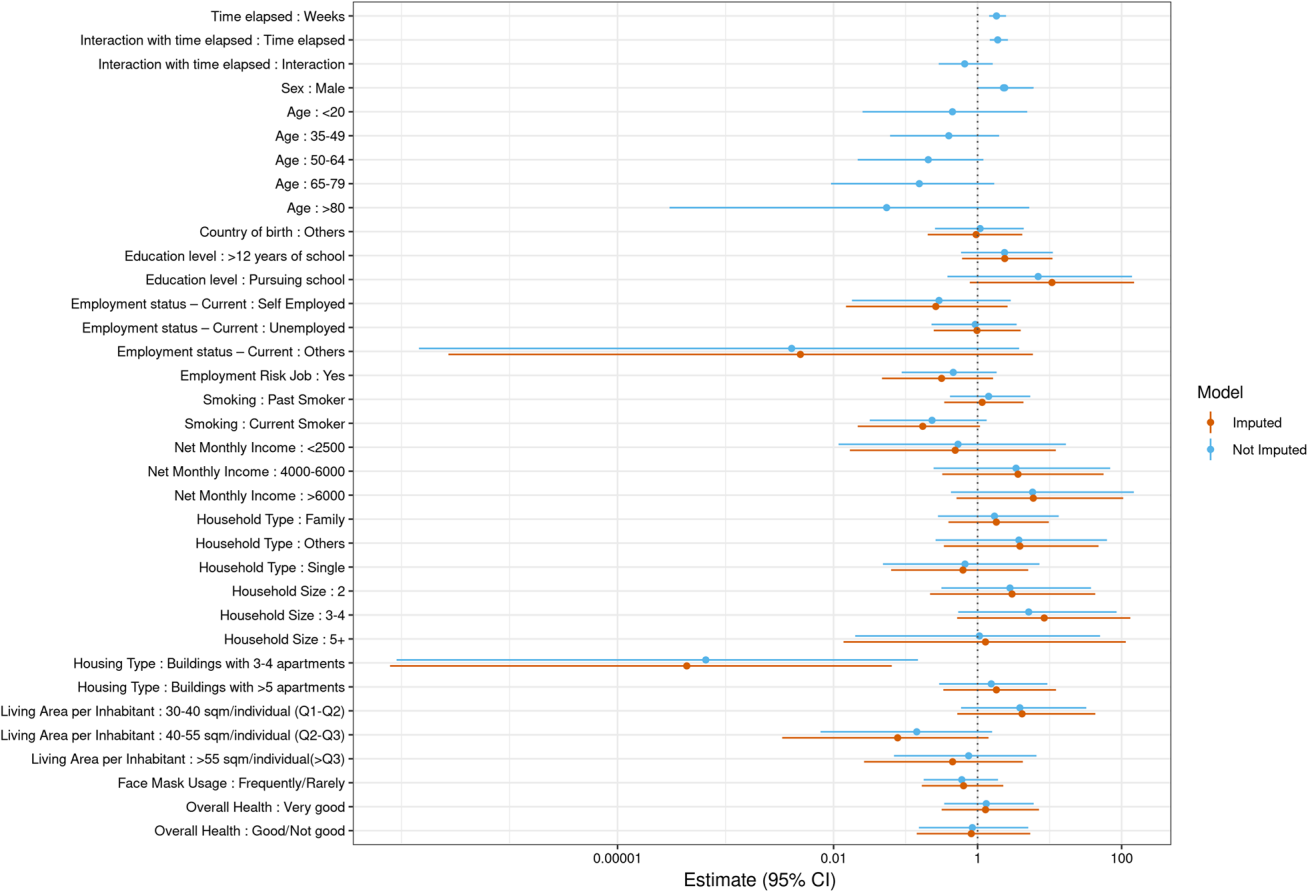
Association between potential risk factors and SARS-CoV-2 sero-positivity taking into account time between 1st and 2nd sampling, baseline result, age and sex. Age and sex was also adjusted for time between 1st and 2nd sampling, baseline result and each other (sex for age and age for sex). Unimputed (blue) and imputed (orange) GLM Models (Bayesian analysis). Main individual level risk factors were time and sex. Odds decreased by age and was lower for participants living in buildings with 3–4 apartments. Changes in the estimates by imputation were small

The distribution of SARS-CoV-2 sero-positivity by covariates is shown in Additional file 1: Table S3. Taking household clustering, time elapsed between baseline and follow-up, and baseline result into account, men had statistically significantly higher odds of sero-positivity at follow-up (OR adjusted for age: 2.4; 95% CI 1.0–6.0; Fig. 5). In addition, SARS-CoV-2 sero-prevalence decreased with increasing age group. It was lower in participants living in small apartment houses compared to participants living in single houses (OR adjusted for age and sex 0.0002; 95% CI 0.0–0.14).

### Household and neighbourhood clustering of SARS-CoV-2 cases

**Fig. 6:**
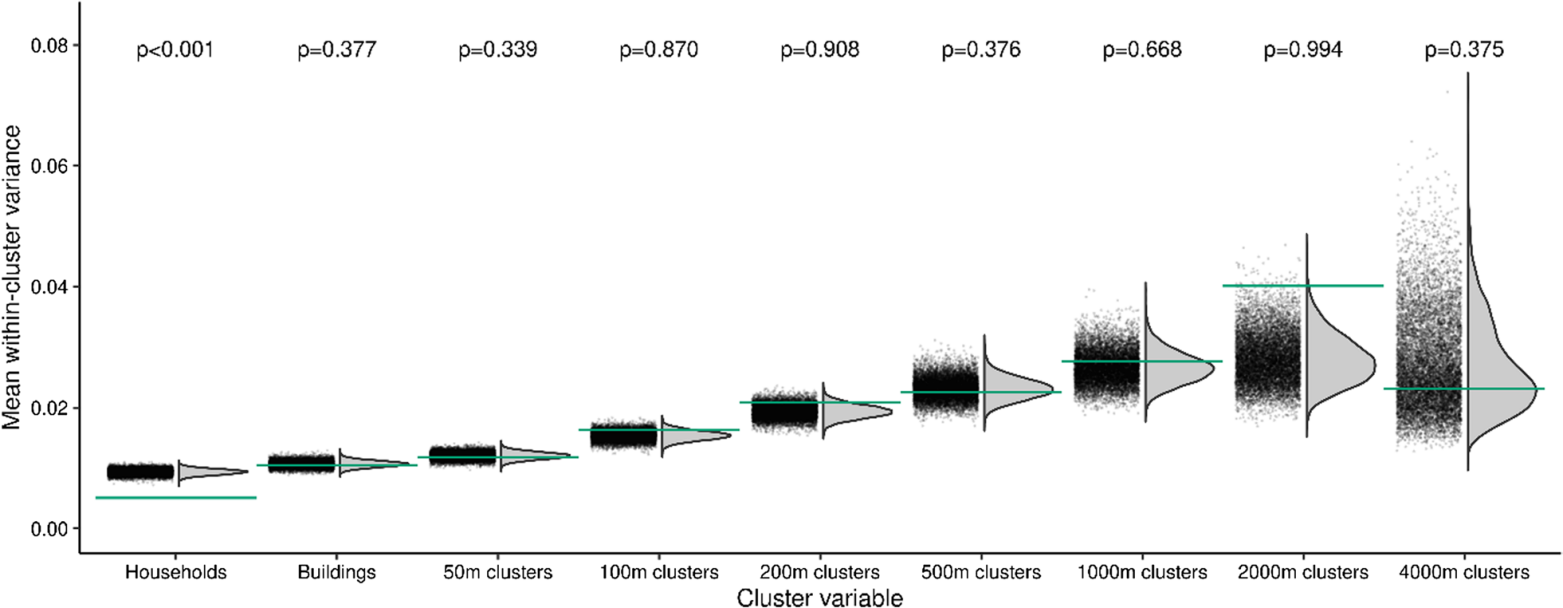
Proximity clustering of test outcomes at 2nd sampling. The grey points and curves show the distribution of mean within-cluster variances for 10,000 random permutations of cluster assignments, the horizontal lines show the observed values. Cluster variables are households, buildings, and geospatial clusters of different sizes. Household membership was left invariant when considering buildings and geospatial clusters. P-values indicate the one-sided probability of observing smaller than observed values under random cluster assignments. Results indicate within household clustering but are not suggestive for neighbourhood transmission

The analysis of potential household and neighbourhood clustering indicated a highly significant within-household clustering of SARS-CoV-2 cases. In contrast, no indications for neighbourhood transmission of SARS-CoV-2 were observed (Fig. 6, Additional file 1: Figure S5).

### Sensitivity analyses on behavioural factors and SARS-CoV-2 sero-incidence

In order to identify which behavioural factors might be related to the different sero-prevalences in men and younger subjects, we compared these factors by sex and age group. The sum of contacts decreased significantly from the two younger age groups to the oldest age group in men and women (p < 0.001; Additional file 1: Table S4). In contrast, the self-estimated health-related risk taking behaviour and leisure time activity level were highest in the age group < 35 years. Comparing men and women, self-estimated health-related risk taking behaviour was statistically higher for men than for women in the age group 35–65 years only (p < 0.001, Additional file 1: Table S5). In contrast, sum of contacts and number of leisure time activities was similar for men and women by age strata (p >  = 0.05).

**Fig. 7:**
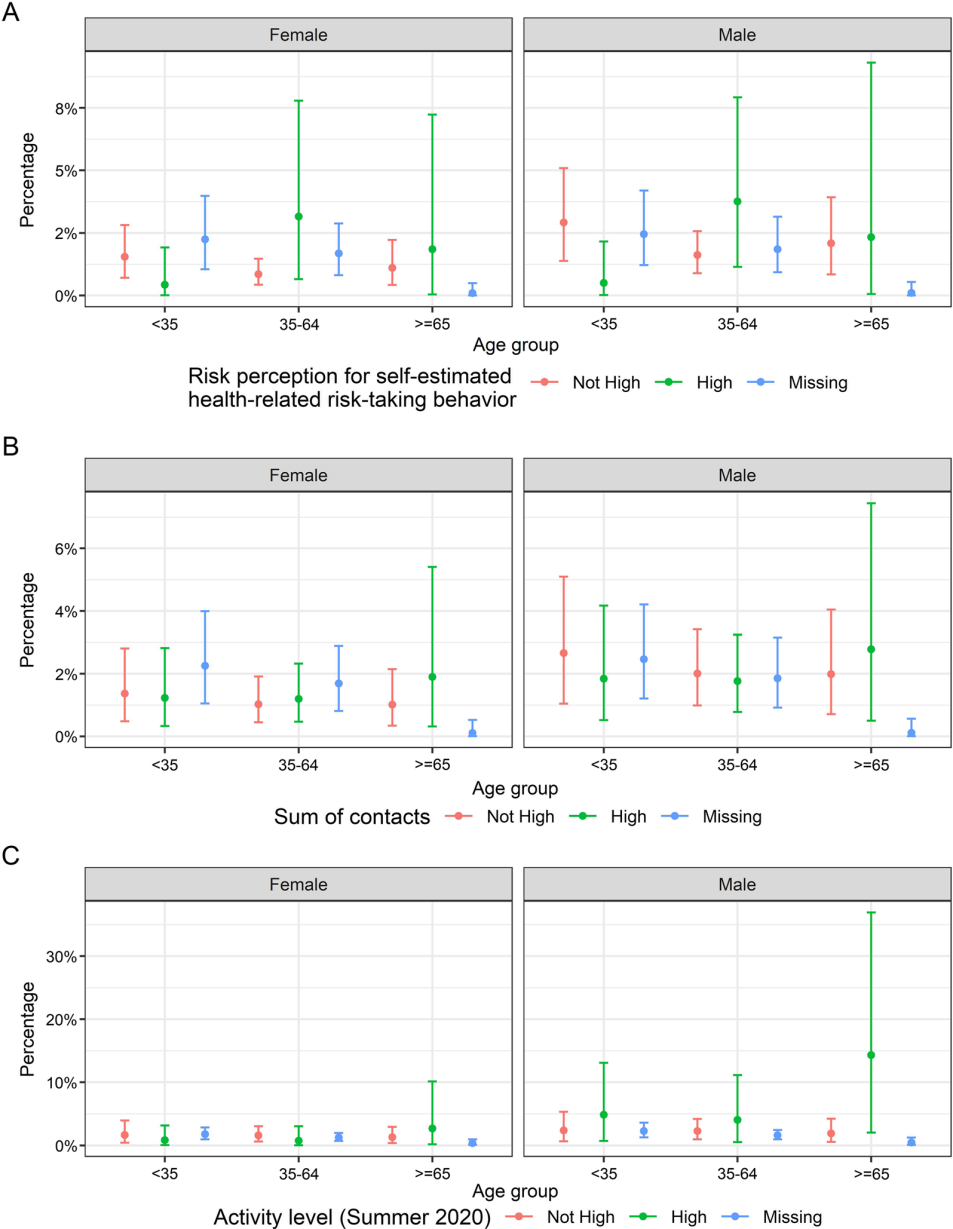
Sero-incidence of SARS-CoV-2 between baseline and follow-up by **A** self-estimated health-related risk-taking behaviour, **B** sum of contacts and **C** leisure time activities in summer 2020 stratified for sex and age group. Results suggest some role of behavioural factors in the risk of SARS-CoV-2 sero-positivity but differences are not statistically significant

In order to check for effect modification by sex, age and behavioural factors, we calculated the sex- and age-stratified SARS-CoV-2 sero-incidence between baseline and follow-up by self-estimated health-related risk taking behaviour (Fig. 7A), number of contacts outside own household (Fig. 7B), number of leisure time activities in summer 2020 (Fig. 7C). These data indicate a slightly higher risk of infection among men and women above the age of 34 years who indicated to have a high health-related risk taking behaviour compared to those with no high health-related risk taking behaviour. Men and women above the age of 64 years showed a higher SARS-CoV-2 sero-incidence if they had more personal contacts compared to participants having fewer personal contacts. Men with more leisure time activities in summer 2020 had a higher SARS-CoV-2 sero-incidence compared to less active men. However, all confidence intervals largely overlapped.

## Discussion

Our data indicate a low SARS-CoV-2 sero-prevalence for the Munich general population living in private households eight months after the start of the pandemic. The incidence between the end of the first wave and the middle of the second wave was about as high as the related number of infections acquired during the first wave. Almost all sero-positive participants at baseline remained sero-positive at follow-up, indicating a high validity of the antibody test. Additionally, this supports previous reports suggesting that the humoral SARS-CoV-2 immune response is stable at least over the first eight months after infection [23, 24]. We also showed a predominance in SARS-CoV-2 sero-positivity among male compared to female participants, and a reduced antibody prevalence with increasing age group.

Based on our data, the sero-prevalence for the Munich population above the age of 13 years living in private households was 3.6% (95% CI 2.9–4.3%). Until the end of November 2020, a total of 30,180 SARS-CoV-2 cases were officially registered in Munich (https://www.muenchen.de/rathaus/Stadtinfos/Coronavirus-Fallzahlen.html#Fallzahlen; Access date: 19-April-2021) which results in a population prevalence of 1.9%. This prevalence increased to 44,377 registered cases by the end of December 2020 (population prevalence 3.0%). The data are not directly comparable, as the official data also include children and persons living in institutions. While the prevalence of infection in children was at that time considered to be smaller than in adults, it was unknown for people living in institutions (e.g., homes for the elderly). Nevertheless, the comparison gives an indication that the percentage of officially registered infections improved considerably compared to the beginning of the pandemic. In a previous publication we estimated that solely one out of four infections was registered by the official infectious diseases surveillance system [3]. A few population-based SARS-CoV-2 sero-studies have been conducted since the beginning of the pandemic (for review see [2]), most of them reporting sero-prevalences during or after the first wave. Up to now, only the Spanish national study reported the results of their follow-up data [25] with a sero-prevalence at follow-up (November 2020) of 5%, and thus comparable to our results.

In our study, one predictor of change in sero-prevalence from baseline to follow-up was male sex. While a higher risk of more severe COVID-19 among men was confirmed in several studies [26], findings on sex-differences in sero-prevalence are still inconsistent [2]. As younger age was also related to a larger increase in SARS-CoV-2 sero-prevalence at follow-up, one might assume that differences in behaviour may contribute to these findings. We could confirm differences in health-risk taking behaviour, frequency of leisure-time activities, and number of contacts outside the own household especially by age. In the stratified analyses of the incidence of infection by age and sex, we observed a tendency that behaviour is related to higher sero-incidence of infection; although the low incidence and the reduced number of respondents to the questionnaires limited the statistical power of these analyses and result interpretation has to be done cautiously. However, the hypothesis that specific behaviour, i.e., restriction of contacts, might reduce the risk of infection is also supported by our observation that patients with autoimmune disease were at reduced risk of SARS-CoV-2 sero-positivity. This finding is in line with studies among, e.g., patients with inflammatory bowel disease [27]. Overall, our findings support the notion that behavioural factors contribute to the spread of the pandemic, and therefore actions to increase adherence to public-health measures (such as information campaigns) are crucial especially in a time when acceptance of measures in the general population is faltering.

Our results also confirm the importance of household clustering while no indications for neighbourhood clustering were seen. The former finding is also supported by the observation that participants from higher income households were at non-significantly higher odds of SARS-CoV-2 sero-positivity. As we took into account total household income (not adjusted for number of persons in the household), single households were more likely to be in the lower income category and thus, at lower likelihood of household transmission.

We also saw a non-significant trend for lower odds of SARS-CoV-2 antibodies in smokers compared to non-smokers (OR 0.2; 95% CI 0.02–1.1), confirming results of a meta-analysis [28]. Here, differences were mainly explained by differences in testing behaviour between smokers and non-smokers, which can be excluded in our study. One of the population-based studies published so far also indicated a lower SARS-CoV-2 sero-prevalence in smokers compared to non-smokers [29]. Whether this is a true effect of, e.g., nicotine [30] or vitamin D [31] or result of some form of bias needs to be evaluated in future studies. Of note is also the tendency for higher odds of SARS-CoV-2 in participants pursuing school, however, the wide confidence interval does not permit strong conclusions.

Among the strengths of our study are its population-based, prospective nature in a large number of participants. Such population-based studies help authorities to plan public health measures based on the prevalence of exposure in the population, its spatial distribution and to further identify risk groups [32]. With increasing availability of vaccines, this study design with further follow-ups will help public health authorities to understand the extent and duration of vaccine-induced immunity [33]. We previously showed a high sensitivity and specificity of the Elecsys**®** Anti-SARS-CoV-2 assay (Roche) used in this sero-study [5]. For the follow-up, we developed and carefully validated a semi-automated protocol using self-sampled DBS for SARS-CoV-2 serology [7]. This approach facilitates field work to a very considerable extent and thus, makes studies with a higher frequency of follow-ups more feasible. Acceptance was high in our study population, and the percentage of participants lost to follow-up comparably low.

However, in the analyses we had to take selective participation into account by modelling the underlying non-response mechanism and calibrating the weights. This way, we could reduce attrition bias in our prevalence and incidence estimates. It is common in prospective cohort studies that baseline participants in younger age groups, with migration background and with lower socio-economic status are less likely to participate at follow-up [4]. While typically participants with positive outcome are also more likely to participate in follow-up studies (34), our baseline participants who were SARS-CoV-2 antibody positive were less likely to take part at follow-up. This gives some indication that unknown sero-status motivated at least part of our baseline participants to take part in the study. Once positive sero-status was known to them, they might have lost interest. Further supported is this hypothesis by the fact that less participants were willing to complete the follow-up questionnaires than to take part in the SARS-CoV-2 antibody follow-up. As a consequence, statistical power to analyse the association between behavioural factor and SARS-CoV-2 sero-positivity was limited. Finally, the age group 14 to 19 years is of specific interest given that data on sero-prevalence in this age group is still limited. However, the number of participants in this age group was low (n = 212) and given the still low sero-prevalence at the time of the study, only 9 of them turned positive limiting the power of further analyses in this age group.

## Conclusions

In conclusion, SARS-CoV-2 sero-prevalence in the Munich general population was still low by the end of 2020. Men and younger parts of the population were more likely to be affected. Risk-taking behaviour might be one reason for these differences. Therefore, non-pharmaceutical public health measures are still important.

## Supplementary Information

**Additional file 1: Figure S1.** For sensitivity and specificity adjusted (left) and unadjusted (right) SARS-CoV-2 sero-prevalence over the follow-up period excluding DBS intermediates. The 95% confidence intervals for the weekly sero-prevalence are based on the 2.5 and 97.5 percentiles from 5,000 repetitions of a cluster bootstrap that accounts for within household clustering. The estimates do not account for sample weights. **Figure S2.** Comparison of the sero-positivity over the follow-up period accounting for sampling weights. **Figure S3.** Sero-prevalence estimates across the different boroughs of Munich using CAR model priors with a single level of spatial autocorrelation as random effects. **Figure S4**. Sero-prevalence estimates across the different boroughs of Munich with CAR model priors through the dissimilarity of the population density in neighbouring boroughs for spatial autocorrelation. **Figure S5.** Distribution of mean within-cluster variance of test results under 10,000 random permutation of cluster assignments, with clusters being households, buildings, and geospatial clusters of different sizes. Household membership left invariant for building and geospatial clusters. Left: Value distribution. Right: 50%, 95%, 99% CIs. Black lines (left) and dots (right) indicate the observed values. **Table S1.** Comparison of the follow-up plasma results, using DBS or Venous Blood Samples. **Table S2**. Comparison of the estimates for the covariate of population density at the constituency level using the Poisson and the negative binomial model. **Table S3.** Course of SARS-CoV-2 antibody status within the KoCo19 follow-up participants. **Table S4.** Summary across age group and sex for the response to the behaviour questionnaires stratified by sex. p-values are from Pearson’s Chi-squared test with simulated p-value (based on 10000 replicates). **Table S5.** Summary across age group and sex for the response to the behaviour questionnaires stratified by age group. p- values are from Pearson’s Chi-squared test with simulated p-value (based on 10000 replicates).

## Data Availability

Our data are accessible to researchers upon reasonable request to the corresponding author taking data protection laws and privacy of study participants into account. To facilitate reproducibility and reuse, the analysis and figure generation code has been made available on GitHub (https://github.com/koco19/epi2) and has been uploaded to ZENODO (https://doi.org/10.5281/zenodo.4707037) for long-term storage.

## Abbreviations

95% CI: 95% Confidence interval
KoCo19: Munich COVID-19 cohort
LMU: Ludwig-Maximilians-University
OR: Odds ratio
ORCHESTRA: Connecting European Cohorts to Increase Common and Effective Response to SARS-CoV-2 Pandemic
SARS-CoV-2: Severe acute respiratory syndrome coronavirus type 2
Yrs: Years
